# Value of eight-amino-acid matches in predicting the allergenicity status of proteins: an empirical bioinformatic investigation

**DOI:** 10.1186/1476-7961-7-9

**Published:** 2009-10-29

**Authors:** Rod A Herman, Ping Song, Arvind ThirumalaiswamySekhar

**Affiliations:** 1 Dow AgroSciences LLC, 9330 Zionsville Road, Indianapolis, IN 46268, USA

## Abstract

The use of biotechnological techniques to introduce novel proteins into food crops (transgenic or GM crops) has motivated investigation into the properties of proteins that favor their potential to elicit allergic reactions. As part of the allergenicity assessment, bioinformatic approaches are used to compare the amino-acid sequence of candidate proteins with sequences in a database of known allergens to predict potential cross reactivity between novel food proteins and proteins to which people have become sensitized. Two criteria commonly used for these queries are searches over 80-amino-acid stretches for >35% identity, and searches for 8-amino-acid contiguous matches. We investigated the added value provided by the 8-amino-acid criterion over that provided by the >35%-identity-over-80-amino-acid criterion, by identifying allergens pairs that only met the former criterion, but not the latter criterion. We found that the allergen-sequence pairs only sharing 8-amino-acid identity, but not >35% identity over 80 amino acids, were unlikely to be cross reactive allergens. Thus, the common search for 8-amino-acid identity between novel proteins and known allergens appears to be of little additional value in assessing the potential allergenicity of novel proteins.

## Background

The use of biotechnological techniques to introduce novel proteins into food crops (transgenic or GM crops) has motivated investigation into the properties of proteins that favor their potential to elicit allergic reactions. Allergy is an atypical detrimental immune response to proteins that are otherwise harmless, and is typically mediated by IgE antibody binding. Thus far, no single property of a protein is known to predict allergenic potential. For this reason, a weight-of-evidence approach to predicting allergenic risk has been adopted which considers multiple factors. These factors include the source of the protein, prevalence of the protein in the transgenic crop, resistance to heat and digestion, and structural similarity to known allergens [1-3].

If a transgenic protein is isolated from a source organism that causes allergy, it is possible that an allergenic protein from the source organism could be inadvertently transferred to the transgenic crop. In this case, IgE antibody binding can be evaluated using serum from patients that are allergic to the source organism to determine if the transgenic protein is an allergen from that source. The intestines are considered the major site of absorbance for allergenic food proteins [4]. Increased titer of a protein in the intestines increases exposure and may favor development of food allergy. The prevalence of the transgenic protein in food and its resistance to processing and cooking may affect the amount of protein ingested, and the resistance of the protein to digestive processes, especially pepsinolysis in the stomach, will affect the amount of protein reaching the intestinal mucosa. Finally, it is possible that structural similarities between the transgenic protein and an existing allergen will be sufficient to allow IgE antibodies in patients sensitive to an existing allergen to cross-react with the transgenic protein causing allergic symptoms.

The methods for evaluating several of these properties of allergens have been questioned. Measuring heat stability based on maintenance of biological activity or polyclonal-antibody binding has been criticized as not being pertinent to destruction of epitopes to which IgE antibodies bind, and empirical evidence that reactions to allergens can actually increase after heating has been reported [5,6]. The prediction of digestibility using *in vitro *simulated gastric fluid assays with purified proteins has also been found to lack significant predictive value [4,7-9].

In the area of bioinformatics, two criteria for evaluating structural similarities between novel food proteins and known allergens are currently used based on amino acid sequence alignments [1-3]. The first criterion is a search over 80-amino-acid stretches (sliding window search) to detect >35% identity between a query protein and known allergens. The window size of 80 amino acids was selected to correspond with a typical domain size in a protein, and recognizes that single protein domains may contain epitopes that mediate antibody binding. The second criterion involves evaluating short amino-acid stretches for identity between the query protein and known allergens. Window sizes of 6 to 8 amino acids have been suggested based on hypothetical epitope sizes, however, use of window sizes of less than 8 amino acids have been largely abandoned based on the high probability of random alignments that are of no predictive value [10,11]. The use of any short-alignment criteria for predicting the allergenic potential of proteins has also been recently criticized [12-14].

Here, we investigated the additional value of searching for 8-amino-acid sequence matches when combined with the criterion of >35% identity over 80 amino acids using the Food Allergy Research and Resource Program (FARRP) allergen database administered by the University of Nebraska, Lincoln [15]. Specifically, we compared each amino-acid sequence in the database, pair-wise, with all other sequences in the database using each criterion. Protein pairs only detected by the 8-amino-acid identity criterion, but not the >35% identity over 80-amino-acids criterion were identified, and these protein pairs were evaluated for relevance to allergenic cross reactivity. These results were used to empirically evaluate the additional value that the 8-amino-acid criterion provides to the allergenicity assessment of novel food proteins.

## Methods

The FARRP AllergenOnline Version 9 database of allergens (released January 2009) was used for all bioinformatic analyses . Each protein was individually removed from the 1,386 allergen database and used as the query protein to assess each of two different criteria compared with each of the remaining 1,385 allergens. The first criterion looked for >35% identity over a sliding window of 80 amino acids using the FASTA (version 34t26) algorithm with the default settings for search parameters (BLOSUM 50, *ktup = 2*, gap penalties = -10/-2). The second query looked for contiguous eight-amino-acid matches between the query sequence and each remaining sequences in the allergen database. Both types of searches are available and explained on the AllergenOnline web site [15]. As with the AllergenOnline web site, the algorithm used for identifying >35% identity over a sliding window of 80 amino acids incorporated an algorithm to account for alignment regions of less than 80 amino acids. If the alignment region (including gaps) was at least 80 amino acids long, then identity of >35%, as indicated by the FASTA output, was used to designate a match. If the alignment region (including gaps) was less than 80 amino acids long, then the number of amino acid matches was divided by 80, and percentages of >35% were considered matches. Unique pairs of homologous proteins meeting each criterion were tabulated, and those meeting only the eight-amino acid criterion but not the >35% identity over 80-amino-acid criterion were identified.

Protein sequences of fewer than 29 amino acids cannot show greater than 35% identity over 80 amino acids since 28 is exactly 35% of 80. Protein sequences from 29 to 79 amino acids almost always require the adjustment to 80 amino acids as described above (except where gaps in the FASTA alignment extend the homology region to over 80 amino acids). Where one member of a protein pair was from 29 to 79 amino acids, the cumulative number of protein pairs meeting only the eight-amino-acid criterion, but not the >35% identity over 80-amino-acid criterion, was plotted against the number of amino acids in the shorter protein in each 8-mer-only pair (cumulative number of pairs as the amino-acid length of the shorter member in each pair decreased). Based on the observed pattern of the plotted data, linear regression was conducted over data from 29 to 40 amino acids, and for points from 39 to 79 amino acids.

Where the shorter protein sequence in each pair was from 39 to 79 amino acids in length, the amino-acid length of the shorter member of each protein pair was compared with the length of a typical full-length protein isoform to determine if the FARRP entry was a complete or partial sequence. For protein pairs meeting only the 8-mer-identity criterion and having ≥80 amino acids for both members of each pair, the FASTA alignment for each pair was examined.

## Results and Discussion

### Results

**Figure 1:**
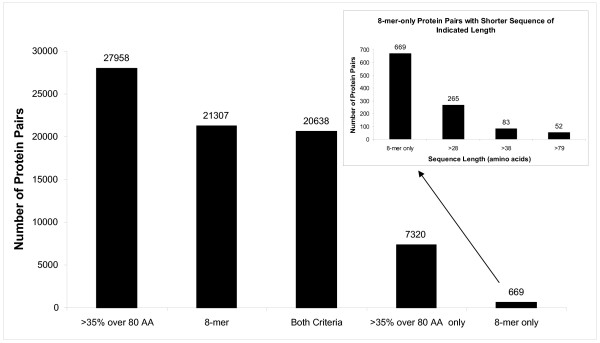
**Number of matching pairs of proteins from the FARRP allergen database that meet the indicated identity criterion**. Inset shows a further breakdown in the number of pairs meeting only the 8-mer criteria where the amino acid length of both proteins in the pair is above a certain amino acid length.

Using each of the 1,386 protein sequences in the FARRP database as a query protein, 959,805 pair-wise comparisons were made. There were 27,958 unique protein pairs that shared >35% identity over 80 amino acids and 21,307 unique protein pairs that shared identical 8-amino-acid contiguous stretches (Figure 1). A total of 669 unique protein pairs met the eight-amino acid criterion but not the >35% identity over 80-amino-acid criterion. Of these 669 protein pairs, 404 (60%) contained at least one protein that was less than 29 amino acids in length making it impossible for the pair to share >35% identity over 80 amino acids (100*28/80 = 35%). Of the protein sequences where both members of the pair were ≥80 amino acids in length, 52 pairs were identified that only met the 8-amino-acid contiguous match criterion [see Additional file 1]. These 52 pairs consisted of 17 pairs of source organisms due to multiple isoforms of each protein being present in most cases.

**Figure 2:**
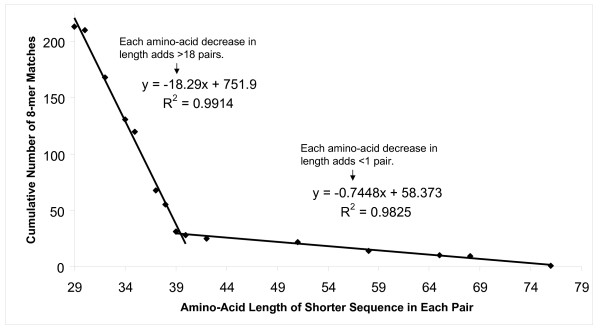
**Effect of Sequence length on 8-mer-only hits for pairs with one protein under 80 amino acids**. Relationship between amino-acid length and the cumulative number of 8-mer-only matches produced. Lines depict the linear regression of proteins from 29 to 40 amino acids and from 39 to 79 amino acids in length.

For protein pairs having the shorter protein in each pair from 29 to 79 amino acids in length, 213 pairs having only 8-amino-acid sequence identity were identified. A plot of the length of the shorter protein in each pair versus the cumulative number of pairs yielded a pattern that was well fit by two individual linear regression lines (Figure 2). There was a natural split in the slope of the line at the 39-amino-acid length. When the shorter protein in the pair was less than 39 amino acids long, each amino-acid reduction resulted in >18 new pairs of proteins sharing only 8 identical contiguous amino acids. For proteins from 39 to 79 amino acids in length, removal of each amino acid increased the number of pairs sharing only 8 identical contiguous amino acids by less than 1. When the number of 8-mer-only pairs was adjusted for the number of proteins in each amino-acid-length category that was present in the FARRP database, the number of pairs generated per protein was still over 10-fold higher for the shorter protein class [see Additional file 2]. It is noteworthy that for proteins ≥80 amino acids, the rate of 8-mer-only pairs was only 4 per 100, and for those proteins less than 29 amino acids in length, the rate of 8-mer-only pairs was 539 per 100. For 8-mer-only pairs with the shorter protein containing 39 to 79 amino acids, 31 unique protein pairs were identified [see Additional file 3]. These 31 pairs of proteins consisted of 17 pairs of source organisms due to multiple isoforms of each protein being present in most cases.

It seems reasonable to conclude from these patterns that the major contributor to the observation that shorter proteins generate more 8-mer-only pairs is the decreased capacity of shorter sequences to share >35% identity over 80 amino acids, rather than a greater propensity to share 8 identical contiguous amino acids with other sequences in the database. Expanding further on this hypothesis, we researched the typical full-length amino-acid length of 8-mer-only pairs where the shorter sequence in each pair was from 39 to 79 amino acids in length. In every case, the shorter protein sequence in each pair was only represented by a partial sequence in the FARRP database, and these sequences ranged from 2 to 52% of a typical full-length sequence [see Additional file 3] [16-24]. This observation fit with the expectation that partial amino acid sequences may be insufficient to detect >35% identity over 80 amino acids when in fact such identity might exist if full-length sequences were available.

We then examined the FASTA alignments and 8-mer matches for those 8-mer-only hits where both members of the pair were ≥80 amino acids in length. Of the 52 protein pairs identified, 25 pairs did not have an identical stretch of 8 or more contiguous amino acids within the FASTA alignment, suggesting that the identified short amino-acid matches were unrelated to overall structural similarity between the proteins in these pairs [see Additional file 1]. This is important because, even though IgE-binding epitopes may consist of short contiguous amino-acid stretches, the presentation of two epitopes within the overall structure of a protein is believed to be critical in clinical cross reactivity. For example, Klinglmayr et al. (2009)[25] grafted putative short amino-acid epitopes from the apple allergen Mal d 1 into the analogous regions of the homologous birch pollen allergen Bet v 1 (64% similarity) and saw increased IgE reactivity in patients with clinical apple-pollen cross reactivity. These investigators recognized that the conserved 3-dimentional shape and almost identical secondary structure of Mal d 1 and Bet v 1 were required to elicit a response from the transplanted short contiguous amino-acid epitopes. Thus, the absence of significant homology between protein pairs in the region of identical short amino-acid stretches suggests that these stretches are unlikely to function as epitopes capable of clinical cross reactivity. In addition to falling outside of the FASTA alignment, all 25 pairs of proteins in this group consisted of low-complexity matches. Low-complexity amino-acid stretches have an increased likelihood of generating random matches [26].

The remaining 27 pairs of proteins, each consisting of ≥80 amino acids, contained 8 or more contiguous amino-acid stretches within the FASTA alignment region. Of these, two pairs of proteins, each containing celery Api g4 and barley alpha-amylase inhibitor component Cma, shared only a 9-amino-acid stretch of homology and thus fall into the category of sharing too little overall homology to indicate likely cross reactivity [see Additional file 1]. Seven pairs of 8-mer-only hits, each containing *Aspergillus fumigates *Asp f 8 and timothy grass Phl p 5, Kentucky blue grass Poa p 5, or velvet grass group V allergen, shared a low complexity 9-mer (PAAAGAAAG) that was homologous to an addition two protein pairs, each containing *Penicillium brevicompactum *Pen b 26 and timothy grass Phl p 5 (PAAAGAAA). Similarly, four protein pairs containing 11S globulin isoforms shared a low-complexity 12-mer (QQGQQQGQQGQQ) or 8-mer (QQGQQGQQ) with mustard Bra j 1 or rapeseed Bra n 1. Finally, Mala s 13 from a yeast species shared a very low complexity 8-mer with two profilin isoforms from wheat (GSHHHHHH). As mentioned earlier, low-complexity amino-acid stretches have an increased likelihood of generating random matches [26].

The last ten 8-mer-only pairs (from the 52 pairs containing only proteins ≥80 amino acids) involved the following four pairs of source organisms: turnip-cedar, *Penicillium chrysogenum*-melon, *Aspergillus niger*-melon, and walnut-wheat. To our knowledge, no evidence of clinical cross-reactive allergy between these pairs of source organisms has been reported. However, these protein pairs do share similar functions between the members of the pairs. The serine protease Cuc m 1 from melon is paired with two proteins also in this same functional group; Pen ch 13 from *Penicillium chrysogenum *and Gi289172 from *Aspergillus niger *[27,28]. Similarly, Gi56550550 from cedar and Bra r 2 from turnip likely share chitinase or chitin-binding functionality [29]. Finally, Jug r 1 from walnut and the low-molecular-weight glutenin from wheat are both seed storage proteins [30,31]. Indeed, the E-scores (significance scores) from the FASTA alignments indicate that many of the pairs of 8-mer-only matches, where both proteins are ≥80 amino acids in length, contain proteins that are likely related evolutionarily [see Additional file 1], although E-scores are not reliable for low complexity matches [26]. It should also be noted that evolutionary or functional relatedness does not imply allergenic cross-reactivity, and both enzymes (such as chitinases and proteases possibly due to their ability to transverse membranes) and seed storage proteins (likely due to their stability and high prevalence in food) are common allergens.

## Discussion

We used the FARRP allergen database of 1,386 amino-acid sequences to investigate the added contribution of 8-mer matches to searches for >35% identity over 80 or more amino acids. We searched the FARRP database using each protein in the FARRP database as the search sequence and found 20,638 protein pairs that met both search criteria, 7,320 that only met the >35%-identity-over-80-amino-acid criterion, and 669 that only met the 8-mer-match criterion (Figure 1). The majority of these latter hits were either too short to meet the >35%-identity-over-80-amino-acid criterion (<29 amino acids, 404 pairs), short enough to be unlikely to meet the criterion (29 to 38 amino acids, 182 pairs), or represented by incomplete and low-complexity amino-acid sequences (39 to 79 amino acids, 31 pairs). The remaining 52 pairs of proteins had substantially complete sequences (≥80 amino acids).

Among the 52 protein pairs having both members over 79 amino acids in length and meeting only the 8-mer criterion, 25 of the short sequence matches were of low complexity and fell outside of the FASTA alignment region, and two pairs had an 8-mer match within a short 9-amino-acid stretch of identity indicating that conformational similarity near the short matches was likely insufficient to present the potential epitopes in a manner that is clinically relevant to cross-reactivity. An additional 15 protein pairs shared low complexity short-sequence matches, suggesting random alignments. The remaining 10 pairs of proteins represented four source organism pairs for which we are aware of no evidence of cross reactivity with respect to allergy.

It has been suggested that matches of short contiguous amino acids adds little to the allergenicity assessment of novel food proteins above that provided by domain-wide or more global homology [12-14]. Some previous work, such as that of Silvanovich et al. and Hileman et al. [10,32], evaluated the frequency of matches generated by the short-contiguous-amino-acid criterion using proteins sequences stored in protein databases (NRAA, nonredundant amino acids, or NRAA1 databases) or coding proteins from rarely allergenic sources (corn, *Zea mays*). These investigations demonstrated high false-positive rates for such searches. Our investigation sought to determine if any truly cross-reactive allergens were uniquely detected by this criterion, and as such, provides a conservative assessment of the value of such searches. This approach predictably resulted in many matches between allergen isoforms; however, this mimics the procedure used for novel proteins and thus seems appropriate.

Our empirical results using protein sequences in the FARRP allergen database are consistent with the previous hypothesis that short contiguous amino acid matches provide little additional value in assessing the potential allergenicity of novel proteins. However, more research is needed to establish that the relatively few pairs of proteins meeting only the 8-mer match criterion are not clinically cross reactive. Further consideration of the value of adding short incomplete sequences to the FARRP database is also recommended since such sequences are of little or no value in searches designed to detect domain-wide or global alignments.

## Conclusion

The current guidelines for conducting allergen homology searches are based on expert opinion rather than experimental evidence [1-3]. Our investigation using the amino-acid sequences of known allergens suggests that short contiguous amino-acid matches alone are a poor predictor of allergenic cross reactivity. The approach taken here may have value in evaluating alternative bioinformatic criteria and may lead to more evidence-based protocols for predicting the cross reactivity between novel proteins and known allergens.

## Abbreviations

AA: amino acid; FARRP: Food Allergy Research and Resource Program; FASTA: fast all.

## Competing interests

The authors are employed by Dow AgroSciences LLC which develops and markets agricultural products, including transgenic crops.

## Authors' contributions

RH and PS collaborated on the conceptualization of the manuscript and PS and AT wrote and conducted informatic searches in support of these concepts. All authors contributed in writing of the manuscript. All authors read and approved the final manuscript.

## Supplementary Material

Additional file 1 **8-mer-only pairs where both proteins are ≥80 amino acids**. Each row contains information for pairs of sequences that are both ≥80 amino acids in length and that share an identical 8-amino-acid stretch, but do not share >35% homology over 80 amino acids. Initial rows in the table show sequence pairs with low-complexity matches falling outside of the FASTA alignment, followed by low-complexity matches within the FASTA alignment. Next are two sequence matches that only share a 9-amino-acid FASTA sequence alignment. Finally, ten complex matches within the FASTA alignment are shown. Click here for file

Additional file 2 **Statistics based on minimum sequence length in 8-mer-only pairs**. Number of FARRP entries, 8-mer-only pairs, and 8-mer-only pairs per protein for four different amino-acid-length classes grouped by the smaller protein in each pair. Click here for file

Additional file 3 **8-mer-only pairs where the shorter protein contains 39 to 79 amino acids**. Each row contains information for pairs of sequences where the shorter sequence is from 39 to 79 amino acids in length and that share an identical 8-amino-acid stretch, but do not share >35% homology over 80 amino acids. Click here for file
