# Supplementary material for: Value of eight-amino-acid matches in predicting the allergenicity status of proteins: an empirical bioinformatic investigation
Source: Clin Mol Allergy. 2009 Oct 29;7:9. doi: 10.1186/1476-7961-7-9 (PMC2773230; doi:10.1186/1476-7961-7-9)
Supplement: Additional file 1 — 8-mer-only pairs where both proteins are ≥80 amino acids. Each row contains information for pairs of sequences that are both ≥80 amino acids in length and that share an identical 8-amino-acid stretch, but do not share >35% homology over 80 amino acids. Initial rows in the table show sequence pairs with low-complexity matches falling outside of the FASTA alignment, followed by low-complexity matches within the FASTA alignment. Next are two sequence matches that only share a 9-amino-acid FASTA sequence alignment. Finally, ten complex matches within the FASTA alignment are shown. [file 1476-7961-7-9-S1.ppt]

## Slide 1
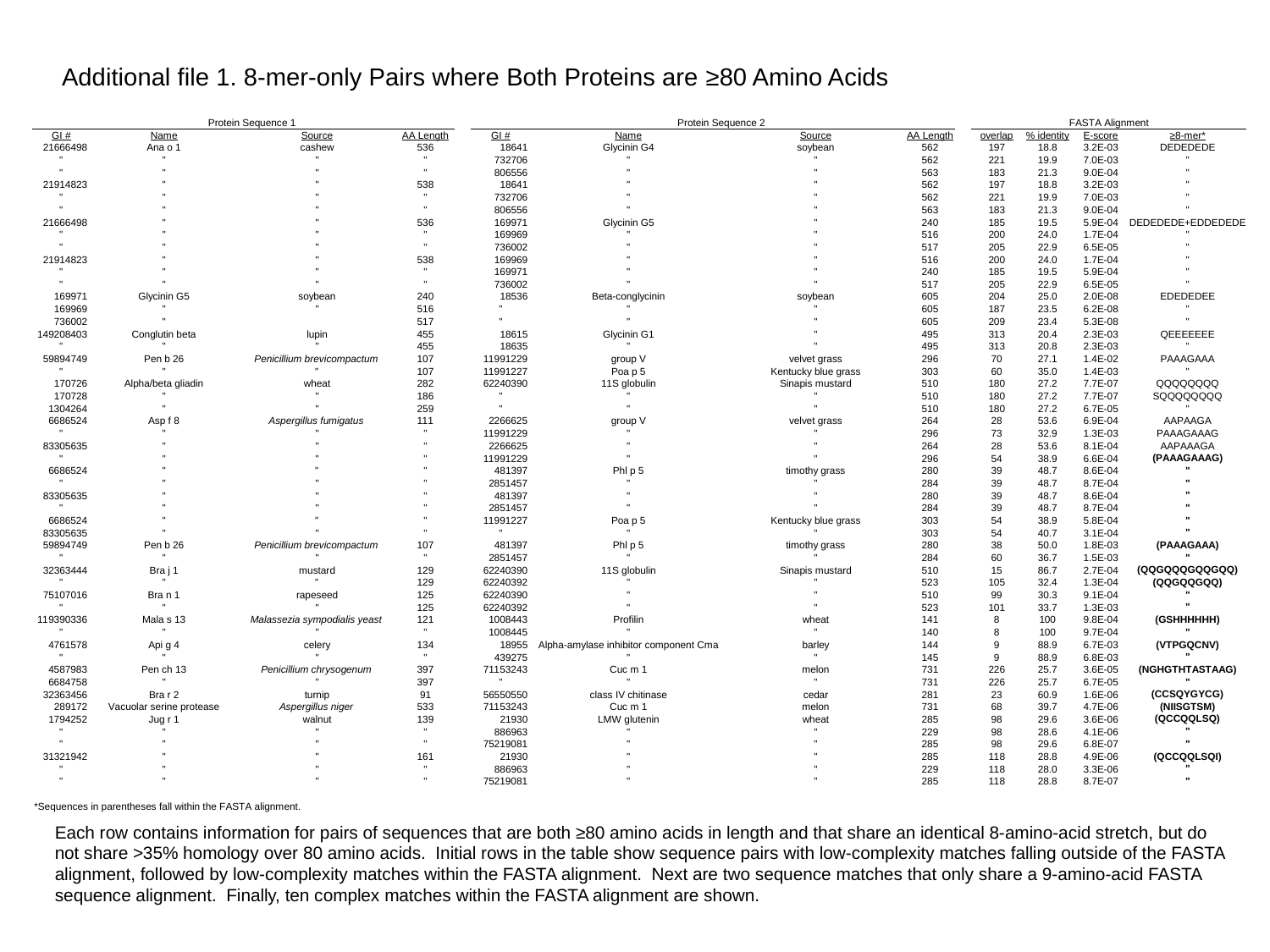

Additional file 1. 8-mer-only Pairs where Both Proteins are ≥80 Amino Acids
Each row contains information for pairs of sequences that are both ≥80 amino acids in length and that share an identical 8-amino-acid stretch, but do not share >35% homology over 80 amino acids. Initial rows in the table show sequence pairs with low-complexity matches falling outside of the FASTA alignment, followed by low-complexity matches within the FASTA alignment. Next are two sequence matches that only share a 9-amino-acid FASTA sequence alignment. Finally, ten complex matches within the FASTA alignment are shown.
