# Supplementary figures and images for: Value of eight-amino-acid matches in predicting the allergenicity status of proteins: an empirical bioinformatic investigation
Source: Clin Mol Allergy. 2009 Oct 29;7:9. doi: 10.1186/1476-7961-7-9 (PMC2773230; doi:10.1186/1476-7961-7-9)

## Slide 1
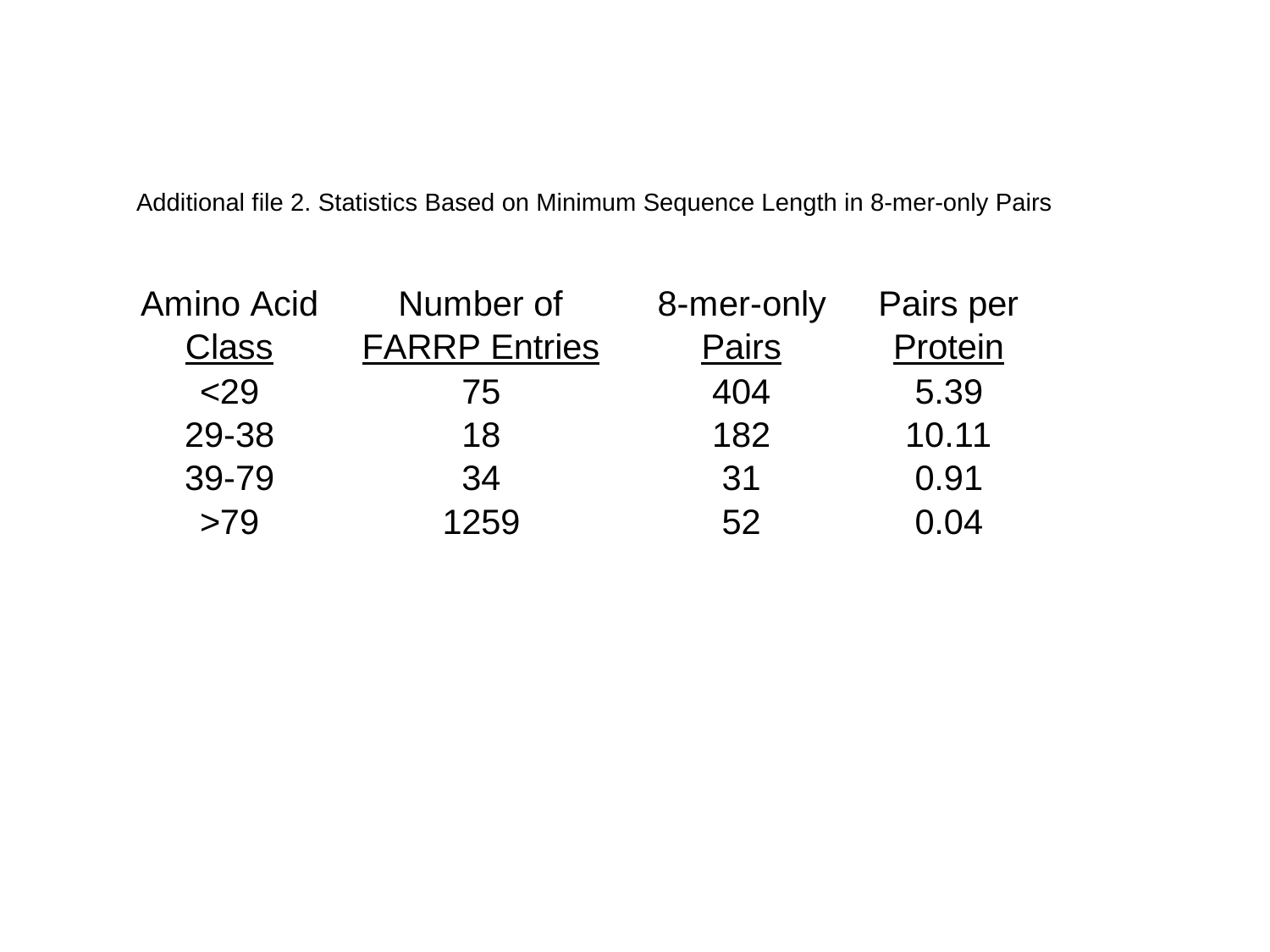

Additional file 2. Statistics Based on Minimum Sequence Length in 8-mer-only Pairs

Supplement: Additional file 2 — Statistics based on minimum sequence length in 8-mer-only pairs. Number of FARRP entries, 8-mer-only pairs, and 8-mer-only pairs per protein for four different amino-acid-length classes grouped by the smaller protein in each pair. [file 1476-7961-7-9-S2.ppt]
