# Supplementary material for: Value of eight-amino-acid matches in predicting the allergenicity status of proteins: an empirical bioinformatic investigation
Source: Clin Mol Allergy. 2009 Oct 29;7:9. doi: 10.1186/1476-7961-7-9 (PMC2773230; doi:10.1186/1476-7961-7-9)
Supplement: Additional file 3 — 8-mer-only pairs where the shorter protein contains 39 to 79 amino acids. Each row contains information for pairs of sequences where the shorter sequence is from 39 to 79 amino acids in length and that share an identical 8-amino-acid stretch, but do not share >35% homology over 80 amino acids. [file 1476-7961-7-9-S3.ppt]

## Slide 1
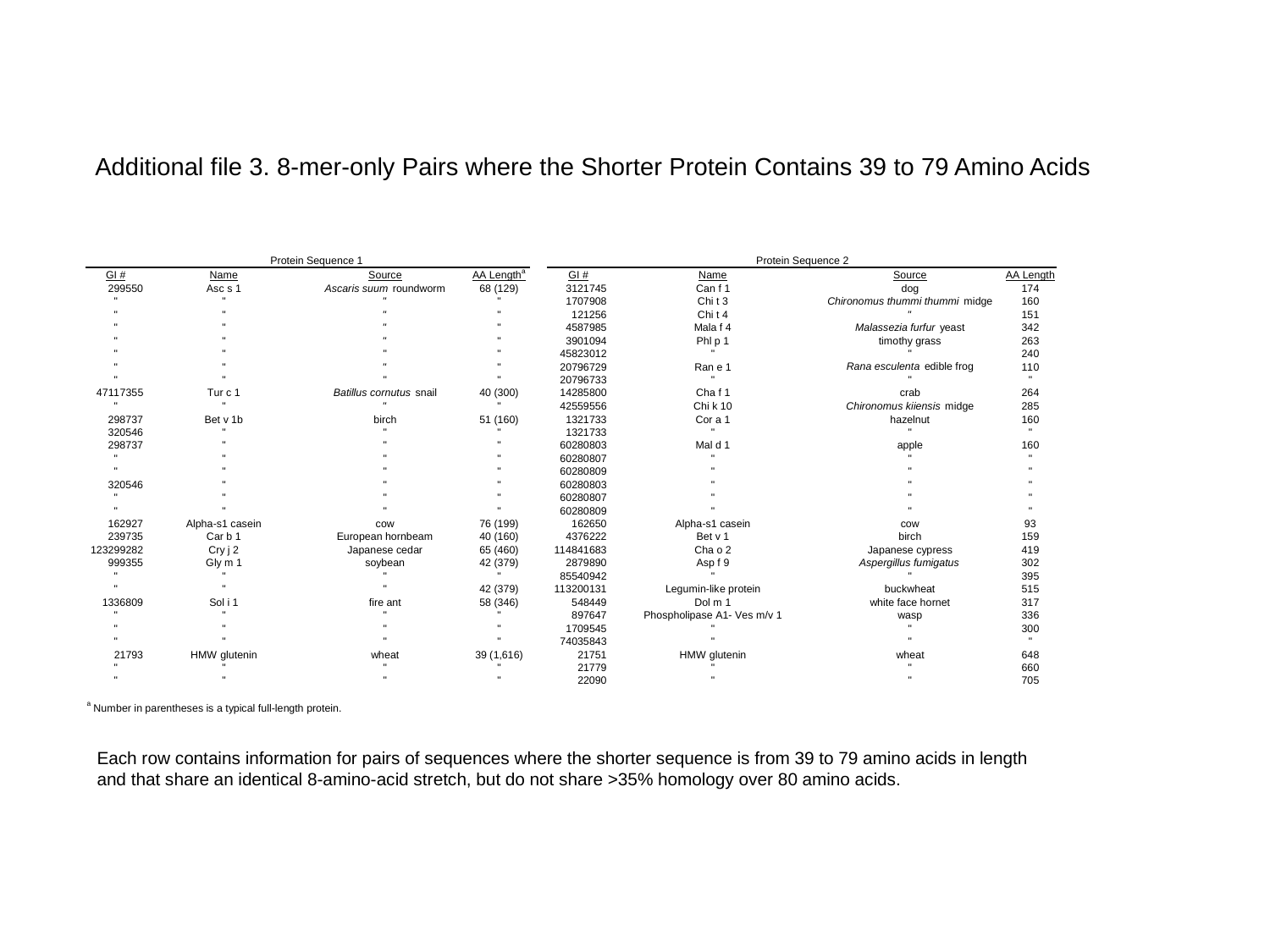

Additional file 3. 8-mer-only Pairs where the Shorter Protein Contains 39 to 79 Amino Acids
Each row contains information for pairs of sequences where the shorter sequence is from 39 to 79 amino acids in length and that share an identical 8-amino-acid stretch, but do not share >35% homology over 80 amino acids.
